# A cyclometalated iridium(III) complex used as a conductor for the electrochemical sensing of IFN-γ

**DOI:** 10.1038/srep42740

**Published:** 2017-02-15

**Authors:** Xiangmin Miao, Chung-Nga Ko, Kasipandi Vellaisamy, Zongbing Li, Guanjun Yang, Chung-Hang Leung, Dik-Lung Ma

**Affiliations:** 1 School of Life Science, Jiangsu Normal University, Xuzhou 221116, PR China; 2 Department of Chemistry, Hong Kong Baptist University, Kowloon Tong, Hong Kong, China; 3 State Key Laboratory of Quality Research in Chinese Medicine, Institute of Chinese Medical Sciences, University of Macau, Macao, China

## Abstract

A novel iridium(III) complex was prepared and used as a conductor for sensitive and enzyme-free electrochemical detection of interferon gamma (IFN-γ). This assay is based on a dual signal amplification mechanism involving positively charged gold nanoparticles ((+)AuNPs) and hybridization chain reaction (HCR). To construct the sensor, nafion (Nf) and (+)AuNPs composite membrane was first immobilized onto the electrode surface. Subsequently, a loop-stem structured capture probe (CP) containing a special IFN-γ interact strand was modified onto the (+)AuNP surface *via* the formation of Au-S bonds. Upon addition of IFN-γ, the loop-stem structure of CP was opened, and the newly exposed “sticky” region of CP then hybridized with DNA hairpin-1 (H_1_), which in turn opened its hairpin structure for hybridizing with DNA hairpin-2 (H_2_). Happen of HCR between H_1_ and H_2_ thus generated a polymeric duplex DNA (dsDNA) chain. Meanwhile, the iridium(III) complex could interact with the grooves of the dsDNA polymer, producing a strong current signal that was proportional to IFN-γ concentration. Thus, sensitive detection of IFN-γ could be realized with a detection limit down to 16.3 fM. Moreover, satisfied results were achieved by using this method for the detection of IFN-γ in human serum samples.

Interferon gamma (IFN-γ) is a type of cytokine that can activate multiple signal transduction pathways through the transcriptional regulation of immunologically relevant genes^1^. IFN-γ is unrestricted by T-helper cells with stimulation by antigens, and is clinically employed to diagnose latent tuberculosis^2^. In addition, dysregulation of IFN-γ secretion is associated with various diseases, such as inflammatory bowel disease, genital herpes simplex virus (HSV) infections and Alzheimer’s disease^3^. Thus, sensitive detection of IFN-γ can be used to investigate the vigor of the immune response as well as for the diagnosis of infectious diseases.

Electrochemical sensing platforms have been extensively used for the detection of biomolecules due of their intrinsic advantages, including good portability, low cost, high sensitivity and simple operation^4^. Recently, numerous electrochemical methods have been developed for IFN-γ detection coupled with a number of signal amplification-based on strategies^1^,^5^,^6^,^7^. For instance, Zhao *et al*. constructed an electrochemical aptasensor for IFN-γ sensing based on the hybridization chain reaction (HCR) coupled with enzyme-signal amplification^8^; Luo and Pu groups realized IFN-γ detection using a dual signal amplification approach by exonuclease-mediated surface-initiated enzymatic polymerization^2^; Wang and He groups developed a sensitive label-free electrochemical aptasensor for IFN-γ detection through nuclease cleavage-assisted target recycling amplification^9^. However, these methods have certain drawbacks, such as the use of enzymes and/or the labelling of probe molecules, which limit the utilization of them for the routine detection of IFN-γ. Thus, developing low-cost, enzyme-free, simple and sensitive strategies for IFN-γ detection continues to attract tremendous interest.

Iridium(III) complexes have been widely used coupled with electrochemiluminescent^10^,^11^,^12^ and fluorescent^13^,^14^,^15^,^16^,^17^ detection techniques due to their high luminescence efficiency, variable photophysical properties and different excited-state characteristics. In addition, such metal complexes can also efficiently intercalate into the grooves of dsDNA strands^18^. Recently, a number of electrochemiluminenscent sensors have been constructed for the sensitive sensing of protein^19^,^20^,^21^ and DNA^22^,^23^. However, to the best of our knowledge, no electrochemical platform has yet been developed by using the iridium(III) complex as an electron conductor for the detection of protein.

In this work, we synthetized an iridium(III) complex that possesses good conductivity, using it as an electron mediator, a sensitive and enzyme-free electrochemical sensor for the determination of IFN-γ based on HCR signal amplification was developed. To construct the sensor, Nf/AuNPs composite membrane was first immobilized onto the electrode surface. After that, a loop-stem structured capture probe (CP) containing a special IFN-γ interact strand was modified onto the (+)AuNP surface *via* the formation of S-Au bonds. Upon addition of IFN-γ, the loop-stem structure of CP was opened, and the newly exposed “sticky” region of CP then hybridized with H_1_ to open the hairpin structure of it, accompanied with the happen of HCR between H_1_ and H_2_ for the formation of dsDNA polymer. Subsequently, iridium(III) complex could bind with the grooves of the dsDNA polymer, producing a strong current signal that was proportional to IFN-γ concentration (Fig. 1). Thus, sensitive IFN-γ detection could be achieved based on identifying the current signal change of differential pulse voltammetry (DPV) signal. Compared to enzyme-assisted signal amplification, HCR avoids the use of enzymes, accordingly improved the robustness and stability of the sensor. Notably, the iridium(III) complex used here could interact in a stable fashion with the unlabelled dsDNA polymers, making the detection of IFN-γ simple and low-cost^24^,^25^.

## Materials and Methods

### Materials

Gold(III) chloride trihydrate (HAuCl_4_·3H_2_O), sodium chloride (NaCl), sodium borohydride (NaBH_4_), bovine serum albumin (BSA), human serum albumin (HSA) and immunoglobulin G (IgG) were obtained from Sigma Aldrich (St. Louis, MO). Interferon gamma (IFN-γ) was purchased from Sino Biological Inc. (China). 20 mM of phosphate-buffered saline (PBS, pH 7.0) was utilized for all of the detection experiments. All other chemicals were analytical grade and utilized without extra purification. Oligonucleotides used in the assay were purchased from Techdragon Inc. (Hong Kong, China) and the DNA sequences of them were shown in Table S1.

### Apparatus

All electrochemical measurements were performed on a CV-3 electrochemical workstation (CH Instruments, Inc. U.S.A.). A conventional three-electrode system including a modified gold electrode, a platinum wire counter electrode and an Ag/AgCl reference electrode was utilized in all of the experiments. Gel electrophoresis was carried out on a GT Mini-Gel system (Bio-Rad Laboratories, Inc., Itally). Size of gold nanoparticles (AuNPs) was detected by using high resolution transmission electron microscopy (HRTEM, Tecnai G2 F20, USA). Nanosurface of the electrode was characterized by scanning electron microscopy (SEM, Hitachi 2000, Japan).

### Synthesis of iridium(III) complex

The structure of the iridium(III) complex [Ir(ppy)_2_(phen-dione)]PF_6_ was shown in Figure S2a, and it was synthetized according to the literature method^21^. A suspension of [Ir(ppy)_2_]_2_Cl_2_ (0.2 mmol) and phen-dione (0.42 mmol) in a solvent solution of mixed CH_2_Cl_2_:MeOH (1:1, 20 mL) was refluxed overnight under the protection of N_2_. The reaction solution was then allowed to cool down to 25 °C, and filtered to remove solid dimer that was not reacted. An aqueous solution of NH_4_PF_6_ (excess) was added into the filtrate and the resulting solution was rotary evaporated until crude product was precipitated out. The precipitate was then filtered and washed with several portions of water (2 × 40 mL) followed by diethyl ether (2 × 40 mL). Afterwards, the product was recrystallized by acetone:diethyl ether vapor diffusion to yield the titled compound as a brown solid (Figure S1).

### Preparation of (+)AuNPs

**Figure 2:**
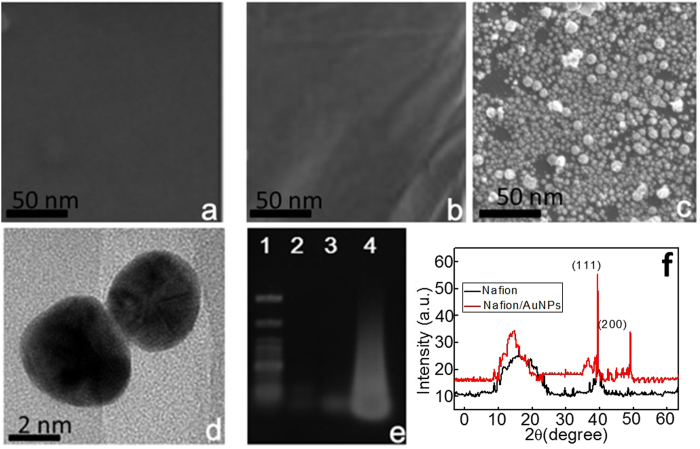
SEM images of bare gold electrode (**a**), Nf modified gold electrode (**b**) and (+)AuNPs/Nf modified electrode (**c**); HRTEM images of (+)AuNPs (**d**); Gel electrophoresis (lane 1: DNA marker, lane 2: 1.0 mM H_1_ + 1.0 mM H_2_, lane 3: 1.0 mM CP and lane 4: 1.0 mM H_1_ + 1.0 mM H_2_ + 1.0 mM CP (**e**); XRD patterns of Nf and Nf/AuNPs (**f**).

Positively charged gold nanoparticles ((+)AuNPs) were obtained through the reported method^22^ based on the reduction of 15 mL HAuCl_4_ (1.0 mM) by using 2 mL of NaBH_4_ (100 mM) in the presence of 2 mL cetyltrimethyl ammonium bromide (CTAB, 10 mM). The color of prepared (+)AuNPs was orange red and the average sizes of them estimated from high resolution transmission electron microscopy (HRTEM) analysis were about 4 nm (Fig. 2d).

### Fabrication of iridium(III) complex/{H_2_/H_1_}_n_/CP/(+)AuNPs/Nf modified gold electrode

The pretreat and modification of gold electrodes with Nf and (+)AuNPs were conducted according to the previous method^28^. After the immobilization of Nf and (+)AuNPs onto the surface electrodes, they were incubated in CP solution over night at room temperature (RT). Subsequently, the CP/(+)AuNPs/Nf modified electrodes were immersed in 6-mercapto-1-hexanol (MCH, 2.0 mM) for 1.5 h at RT to decrease the nonspecific DNA adsorption and to optimize the orientation of the aptamer. Then, the CP/(+)AuNPs/Nf modified electrodes were incubated in IFN-γ solution (1 h) to open the hairpin structure of CP for the following HCR between H_1_ and H_2_. At last, the {H_2_/H_1_}_n_/IFN-γ/CP/(+)AuNPs/Nf modified electrodes were immersed in PBS and CH_3_CN (8/2, *v/v*) buffer containing 20 μM of iridium(III) complex to realize the interaction of iridium(III) complex with dsDNA polymers.

### Gel electrophoresis

Before conducting the gel electrophoresis, H_1_ and H_2_ samples were heated to 95 °C for 5 min and then allowed to cool down to 25 °C before using. 1.0 wt% agarose gel was prepared for DNA samples loading. After the addition of different DNA samples (4.0 μL, 1.0 μM), gel electrophoresis was performed by using 1.0 × TAE as running buffer at a constant potential of 78 V for 40 min. At last, the gels were photographed by using the gel image system after Stains-All staining by ethidium bromide (EB) solution for 15 min.

### Electrochemical detection of IFN-γ

To construct the electrochemical IFN-γ detection, CP/(+)AuNPs/Nf modified gold electrodes were immersed into PBS (20 mM, pH 7.0) containing different concentration of IFN-γ for 60 min at 37 °C. Then, they were immersed into H_1_ and H_2_ solution (2.0 μM) for 50 min for the happen of HCR at RT. After that, the {H_2_/H_1_}_n_/IFN-γ/CP/(+)AuNPs/Nf modified gold electrodes were immersed into the solution that contained of iridium(III) complex for 90 min to realize the interaction between iridium(III) complex and dsDNA polymers. Finally, the electrochemical characteristics of the sensor were determined in 20 mM of PBS (pH 7.0) through differential pulse voltammetry (DPV) experiments from −0.2 to 0.2 V.

## Results and Discussion

### Characteristics of the modified electrodes

The establishment of (+)AuNPs on the electrode surface can greatly improve the amount of CP immobilization. Here, SEM images were used to monitor the formation of Nf/(+)AuNPs, and an obvious thin film of Nf appeared after 5 μL of 1.0 wt% Nf added onto the gold electrode surface (Fig. 2b) compared to that of the bare gold electrode (Fig. 2a). Then, after the adsorption of (+)AuNPs, a homogeneous nano-surface was observed (Fig. 2c). Moreover, Nf and Nf/AuNPs were investigated using X-ray diffraction (XRD) (Fig. 2f). The XRD pattern of Nf is consistent with cubic phase, referring to the standard spectra (JCPDS 15-0770), which displays two characteristic peaks occurring at 2θ of 17°, 39°. Then, two obvious characteristic peaks of AuNPs at 39.5° and 49° for 111 and 200 appeared after the formation of Nf/AuNPs, and a shift in the diffraction peak of Nf was observed due to its combination with AuNPs.

### Gel electrophoresis of different DNA strands

After the addition of IFN-γ, HCR generated long-chain dsDNA polymers. Thus, gel electrophoresis was performed to verify the happen of HCR. As shown in Fig. 2e, HCR amplification between H_1_ and H_2_ triggered by CP resulted in an obvious high molecular-weight smeared band (lane 4) compared with that for the H_1_/H_2_ mixture (lane 2) and MB (lane 3), and this was consistent with our previous reports^27^.

### Conductivity of different iridium(III) complexes

**Figure 3:**
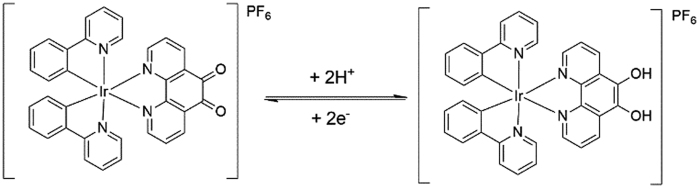
The conductivity mechanism of iridium(III) complex used in our assay.

To monitor the conductivity mechanism of the iridium(III) complex, different iridium(III) complexes were synthesized, and scanned in PBS buffer (pH 7.0). The results in Figure S3 showed that the iridium(III) complex used in our assay (**a**) has an good redox peak while that for others (**b** and **c**) exhibited no obvious peak current (curves b and c). The conductivity of the iridium(III) complex used in our assay could be attributed to the reversible oxidation-reduction reaction between the two ketonic groups and two oxhydryl groups involving the transfer of protons and electrons (Fig. 3).

### Characteristics of different electrodes

**Figure 4:**
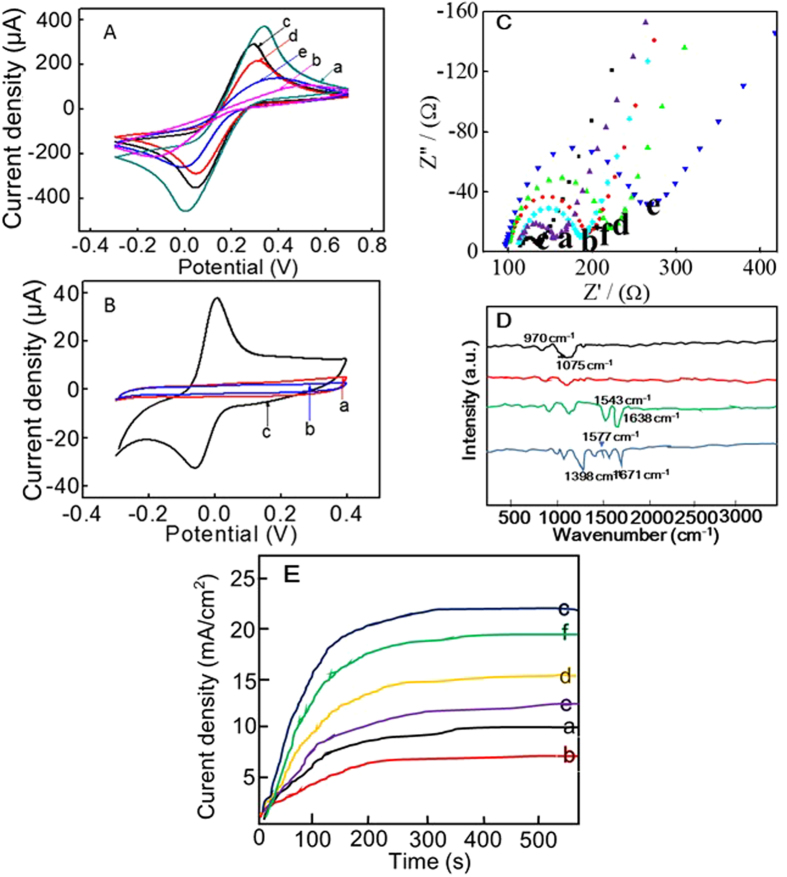
(**A**) CV experiments in Fe(CN)^3−/4−^: (a) bare electrode, (b) Nf, (c) AuNPs/Nf, (d) CP/AuNPs/Nf and (e) {H_2_/H_1_}_n_/IFN-γ/CP/AuNPs/Nf modified electrodes; (**B**) CV experiments in PBS: (a) bare electrode, (b) {H_2_/H_1_}_n_/IFN-γ/CP/AuNPs/Nf and (c) iridium(III) complex/{H_2_/H_1_}_n_/IFN-γ/CP/AuNPs/Nf modified electrodes; EIS (**C**) and chronoamperometric measurements (**E**) in Fe(CN)^3−/4−^: (a) bare electrode, (b) Nf, (c) AuNPs/Nf, (d) IFN-γ/CP/AuNPs/Nf and (e) {H_2_/H_1_}_n_/IFN-γ/CP/AuNPs/Nf modified electrodes; (**D**) FT-IR studies of Nf (a), Nf/AuNPs (b), IFN-γ/CP/AuNPs/Nf (c) and iridium(III) complex/{H_2_/H_1_}_n_/IFN-γ/CP/AuNPs/Nf (d).

The modification process of the electrodes was characterized by using cyclic voltammetry (CV) experiments in the presence of 50 mM Fe(CN)_6_^3−/4−^. As shown in Fig. 4A, a stable and well-defined redox peak was obtained for the bare gold electrode (curve a), and the peak current decreased greatly after the immobilization of Nf due to the character of it that can hinder electron transfer (curve b). Subsequently, an obvious increase of the peak current appeared after the adsorption of (+)AuNPs, because of the good electrical conductivity of (+)AuNPs (curve c). Then, the immobilization of CP onto (+)AuNPs surface based on Au-S bond greatly resulted in a decreased peak current (curve d). The reason for that mainly because MB is negatively charged, which can lead to the electrostatic repulsion with negatively charged [Fe(CN)_6_]^3−/4−^ and hinder the electron transfer between the electrode and [Fe(CN)_6_]^3−/4−^. Moreover, after the happen of HCR between H_1_ and H_2_, the current response of [Fe(CN)_6_]^3−/4−^ redox peak decreased further due to the increase of negatively-charged DNA strands (curve e). Meanwhile, CV experiments were also conducted in PBS solution to demonstrate the interaction of iridium(III) complex with dsDNA polymers. It could be seen from Fig. 4B that a well-fined redox peak appeared after the incubation of {H_2_/H_1_}_n_/IFN-γ/CP/(+)AuNPs/Nf modified gold electrodes with the iridium(III) complex (curve c), which could be attributed to the interaction of the iridium(III) complex with the grooves of the dsDNA polymers. On the contrary, no peaks appeared for the bare gold electrode (curve a) or {H_2_/H_1_}_n_/IFN-γ/CP/(+)AuNPs/Nf modified gold electrodes (curve b) in the absence of iridium(III) complexes, indicating the outstanding conductive capability of the iridium(III) complex.

In addition, electrochemical impedance spectroscopy (EIS) was also used to monitor the fabrication process of the modified electrodes. As shown in Fig. 4C, using 50 mM of Fe[(CN)_6_]^3−/4−^ as the detection solution, a relatively small resistance was observed at the cleaned GCE (diagram a). Then, after the immobilization of the Nf membrane, the resistance gradually increased (diagram b), indicating that Nf inhibited the electron transfer between the solution and the base electrode. Subsequently, an obvious decrease of the resistance appeared after the adsorption of (+)AuNPs, because of the good electrical conductivity of (+)AuNPs (diagram c). However, after the immobilization of CP, IFN-γ, and the initiation of HCR between H_1_ and H_2_, the resistance increased gradually (diagrams d and e). The reason for the increased resistance is mainly due to the fact that the DNA strands are negatively charged, which directly led to electrostatic repulsion with the negatively charged [Fe(CN)_6_]^3−/4−^, hindering the electron transfer between the electrode and [Fe(CN)_6_]^3−/4−^. Finally, after the interaction of the iridium(III) complex with dsDNA polymers, the resistance decreased significantly, indicating that the iridium(III) complex has an outstanding conductive capability (diagram f). Meanwhile, chronoamperometric measurements that constructed in 50 mM of [Fe(CN)_6_]^3−/4−^ at a constant potential of –0.3 V further illustrated the modification process of the electrodes. It could be seen from Fig. 4E that a stable current appeared within 200 s, and a corresponding current change was obtained with the stepwise-modification of the electrode, which was consistent with the EIS results.

Fourier transform-infrared spectroscopy (FTIR) is a useful tool for characterizing the characteristics of different materials. As shown in Fig. 4D, characteristic IR peaks of Nf appeared around 970 cm^−1^ (–COC– linkages), 1075 cm^−1^ (–SO3 H) and 1156 cm^−1^ (–CF_2_–) (curve a)^29^. After the adsorption of AuNPs, an obvious shift of the IR peaks of Nf could be found, indicating the effective adsorption of AuNPs onto the Nf membrane (curve b). Then, two obvious IR peaks appeared around 1543 cm^−1^ (amide II band, C–N stretch coupled with N–H bend) and 1638 cm^−1^ (amide I band, mainly C=O stretch), which displayed the FT-IR spectra of IFN-γ protein (curve “a”) after the immobilization of CP and IFN-γ onto the electrode surface (curve c)^30^. After that, the interaction of the iridium(III) complex directly induced a shift of the peak for the C=O bond, and new peaks of C=C at 1577 cm^–1^ and C-N at 1398 cm^–1^ confirmed the successful interaction of the iridium(III) complex with the dsDNA polymers^31^,^32^.

### Optimization of the experimental conditions

**Figure 5:**
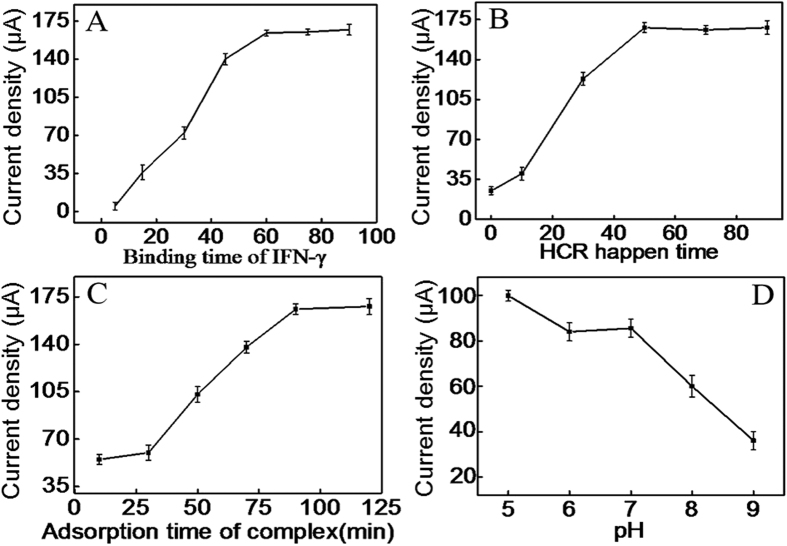
Effect of binding time of IFN-γ (**A**), the HCR time (**B**), the interaction time of iridium(III) complex with dsDNA polymers (**C**) and pH of the buffer (**D**).

The electrochemical signal of the iridium(III) complex mainly based on the amount of it that interacted into the grooves of dsDNA polymers. Thus, the specific bind of IFN-γ with CP can directly affect the happen of HCR, and accordingly affect the interaction of iridium(III) complex. As shown in Fig. 5A, the oxidation peak current of iridium(III) complex enhanced with the increase of the binding time between IFN-γ and CP in the range from 5 to 60 min and then reached a plateau, indicating 60 min could realize the effective binding of CP with IFN-γ. Thus, 60 min was selected as the optimum binding time between IFN-γ and CP.

In addition, the hybridization time between CP and H_1_/H_2_ mixture directly affected the happen of HCR. As displayed in Fig. 5B, the electrochemical signal of iridium(III) complex enhanced with increasing hybridization time of them in the range of 0–50 min. Such result indicated that HCR could effectively happen within 50 min. Therefore, 50 min was chosen as the optimum hybridization time.

The interaction time of iridium(III) complex into the grooves of dsDNA polymers was another crucial parameter that could influence the sensitivity of the detection assay. As displayed in Fig. 5C, the current signal was proportional to the interaction time of iridium(III) complex over the range from 5 to 90 min and then reached a plateau. So 90 min was used for the interaction of iridium(III) complex with dsDNA polymers.

The pH of the PBS also has an effect on the electrochemical behavior of the sensor because the activity of IFN-γ and the oxidation-reduction of iridium(III) complex may be affected by the acidity of the solution^33^. Just as shown in Fig. 5D, the peak current of iridium(III) complex decreased along with the increasing pH value from 5.0 to 9.0 and a plateau from 6.0 to 7.0. Considering that high acid solution may affect the activity of the protein, pH 7.0 was selected as the optimum pH of PBS.

### Sensitivity of the assay

**Figure 6:**
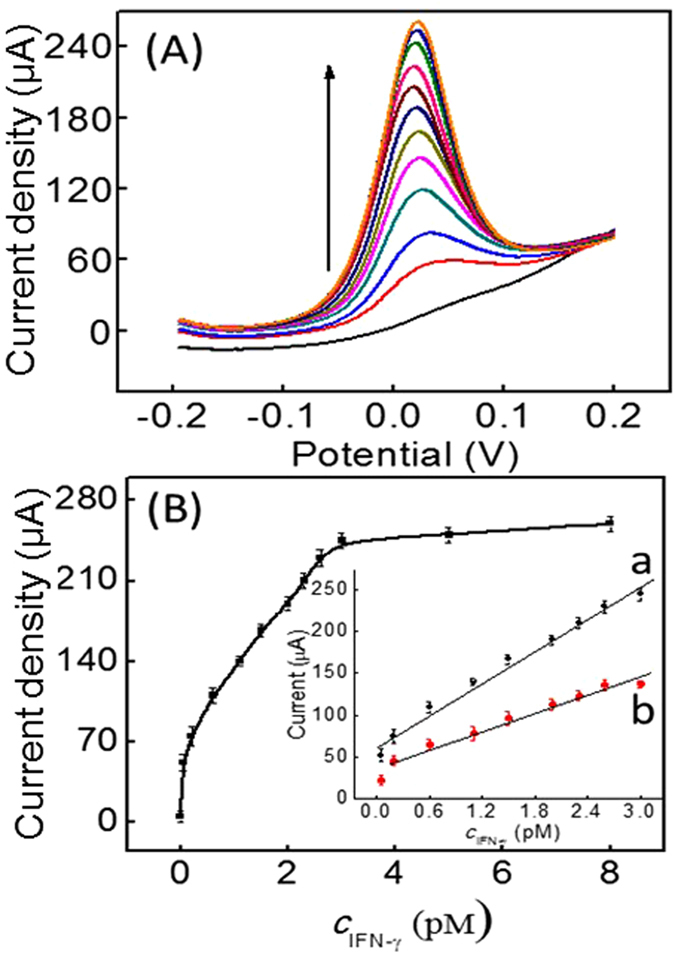
(**A**) DPV experiments for IFN-γ detection in 20 mM of PBS (pH 7.0); (**B**) Dose-response curve and calibration curve (inset) for IFN-γ detection (curve a: the proposed sensor and curve b: the sensor with {H_2_/H_1_}_n_/IFN-γ/CP modification).

Under optimal conditions, IFN-γ detection could be realized based on monitoring the current signal change. As shown in Fig. 6A, the DPV peak currents of the iridium(III) complex increased along with the increase of IFN-γ concentration, and a linear range from 50 fM to 3.0 pM was obtained for IFN-γ detection (Fig. 6B, linear a), with a detection limit of 16.3 fM (3*σ*/slope). The linear regression equation was I = 63.35 + 63.58*c (c*: pM, R^2^ = 0.9971). The detection limit of the proposed method was lower than the reported aptamer-based IFN-γ detection methods (Table S2)^5^,^8^,^9^,^34^,^35^,^36^. Such high sensitivity mainly attributed to the dual signal amplification of HCR and (+)AuNPs. Meantime, we compared the performance of this sensor with a similar sensor {H_2_/H_1_}_n_/IFN-γ/CP that lacked (+)AuNPs. A linear relationship between peak current and IFN-γ concentration was obtained in the range of 200 fM–2.5 pM (Fig. 6B, linear b), illustrating the function of the (+)AuNPs as an electron mediator and signal amplifier.

### Selectivity and stability of the sensor

The selectivity of the sensor was investigated by monitoring the current response of the sensor to IFN-γ, bovine serum albumin (BSA), immunoglobulin G (IgG) and human serum albumin (HSA). As shown in Figure S4, the current signal of iridium(III) complex for 1.5 pM of IFN-γ was greater than 15 pM of various other substances. These results obviously illustrated the high selectivity of the sensor for IFN-γ detection, which was mainly due to the high specificity of CP to IFN-γ. In addition, the stability of the sensor was studied by storing the sensor at room temperature for 10 days, and the sensor kept 95.2% of its initial response, which indicated that the sensor has a good life time for IFN-γ detection.

### IFN-γ detection in human serum samples

Moreover, the application of the sensor for IFN-γ determination in serum samples was evaluated in a detection system containing 5.0% (*v/v*) human serum. As shown in Table S3, a good recovery in the range of 96.6–108.1% was obtained for IFN-γ detection with a relative standard deviation (RSD) between 1.87% and 4.06%, indicating the potential application of the assay for quantitative detection of IFN-γ in complex human samples.

## Conclusion

In conclusion, an enzyme-free and highly sensitive electrochemical sensor for IFN-γ detection was developed by utilizing a novel iridium(III) complex as conductor coupled with a dual signal amplification mechanism involving HCR and (+)AuNPs. Importantly, this amplification mechanism avoids the use of enzymes. Moreover, the iridium(III) complex used here could interact stably with formed dsDNA polymers without modification, making the IFN-γ detection assay simple and low-cost. Finally, the assay was highly selective for IFN-γ due to the specificity of CP for IFN-γ, and could also function in diluted human serum. Thus, this approach could possibly be applied for developing biosensors to monitor IFN-γ levels associated with various human diseases.

## Additional Information

**How to cite this article**: Miao, X. *et al*. A cyclometalated iridium(III) complex used as a conductor for the electrochemical sensing of IFN-γ. *Sci. Rep.* **7**, 42740; doi: 10.1038/srep42740 (2017).

**Publisher's note:** Springer Nature remains neutral with regard to jurisdictional claims in published maps and institutional affiliations.

## Supplementary Material

Supplementary Information

## Figures and Tables

**Figure 1:**
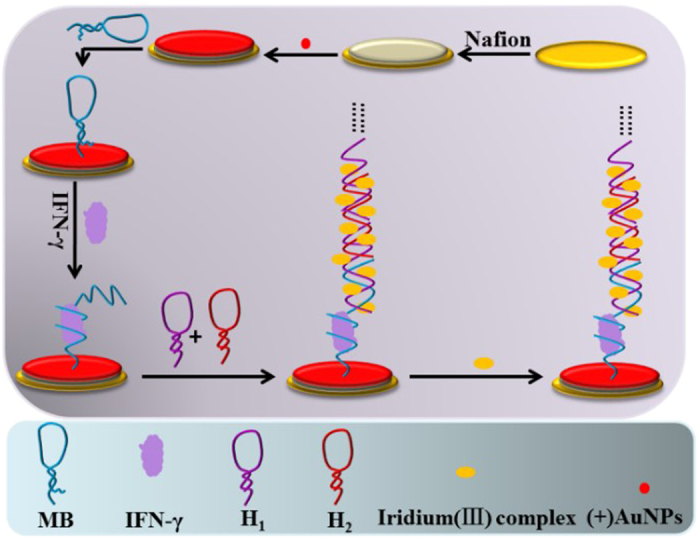
Scheme of the electrochemical IFN-γ detection assay.
