# Supplementary material for: A cyclometalated iridium(III) complex used as a conductor for the electrochemical sensing of IFN-γ
Source: Sci Rep. 2017 Feb 15;7:42740. doi: 10.1038/srep42740 (PMC5309891; doi:10.1038/srep42740)
Supplement: Supplementary Information [file srep42740-s1.pdf]

A cyclometalated iridium(III) complex used as a conductor for the electrochemical sensing of IFN-γ

Xiangmin Miao[a,b,*], Chung-Nga Ko[b], Kasipandi Vellaisamy[b], Zongbing Li[a], Guanjun Yang[c], Chung-Hang Leung[c], Dik-Lung Ma[b,*]

[a]School of Life Science, Jiangsu Normal University, Xuzhou 221116, PR China.

[b]Department of Chemistry, Hong Kong Baptist University, Kowloon Tong, Hong Kong, China.

[c]State Key Laboratory of Quality Research in Chinese Medicine, Institute of Chinese Medical Sciences, University of Macau, Macao, China.

* Corresponding authors:

Dr. Dik-Lung Ma, E-mail: edmondma@hkbu.edu.hk, Tel: (+852) 3411-7075, Fax: (+852) 3411-7348.

Dr. Xiangmin Miao, E-mail: mxm0107@jsnu.edu.cn, Tel: (+86) 516 83403170.

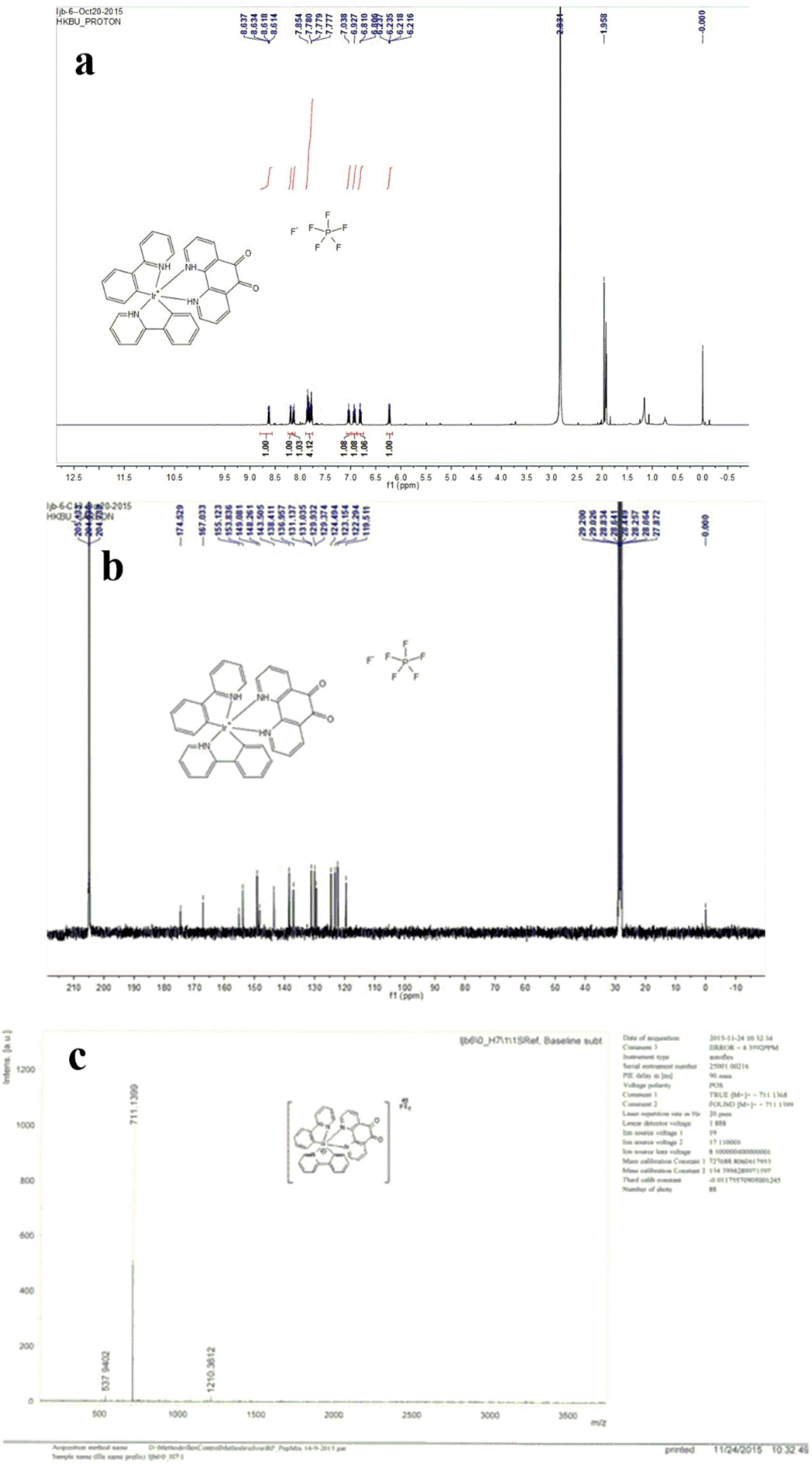

Figure S1. ^{1}H NMR (a), ^{13}C NMR (b) and MALDI-TOF-HRMS (c) of the iridium(III) complex $[Ir(ppy)_2(phen\text{-}dione)]PF_6$.

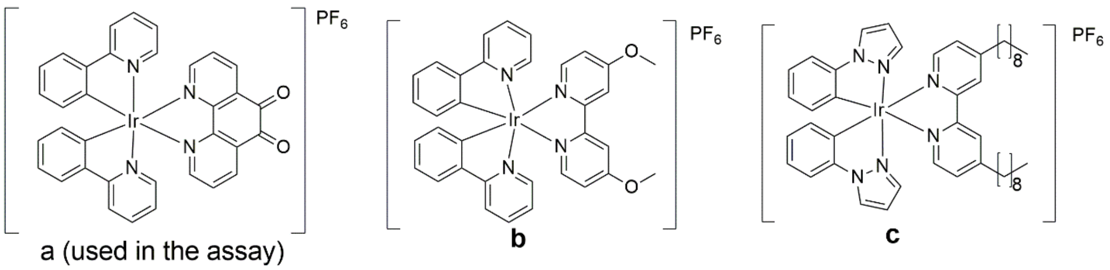

Figure S2. The structure of different iridium(III) complexes. "**a**" was used for sensor preparation. "**b**" was reported in *J. Med. Chem.*, **2015**, *58*, 6694 and "**c**" was reported in *Anal. Chem.*, **2016**, *88*, 981.

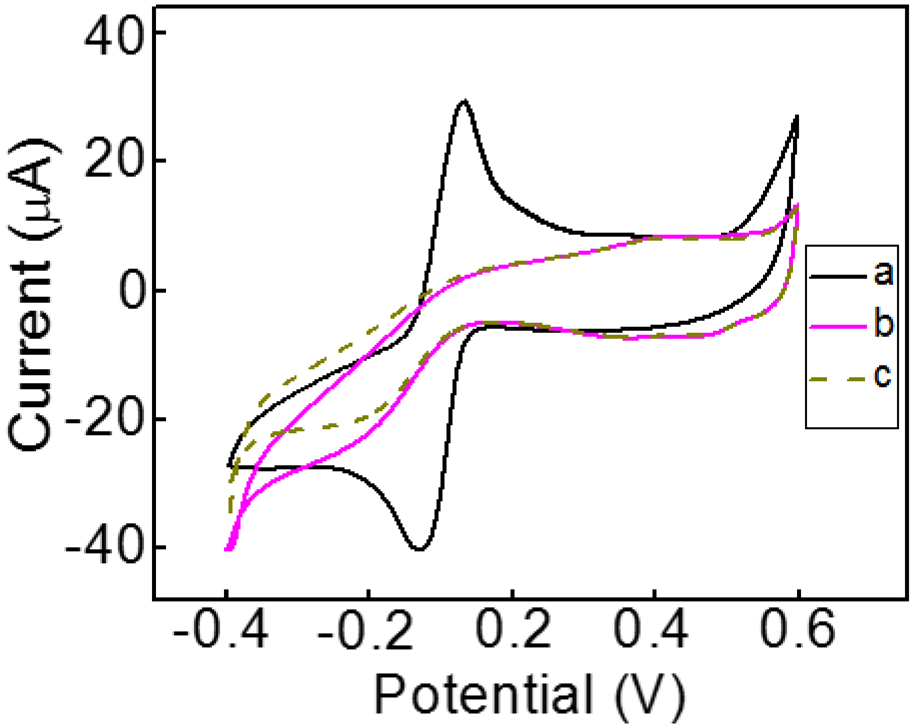

Figure S3. Conductivity of different iridium(III) complexes.

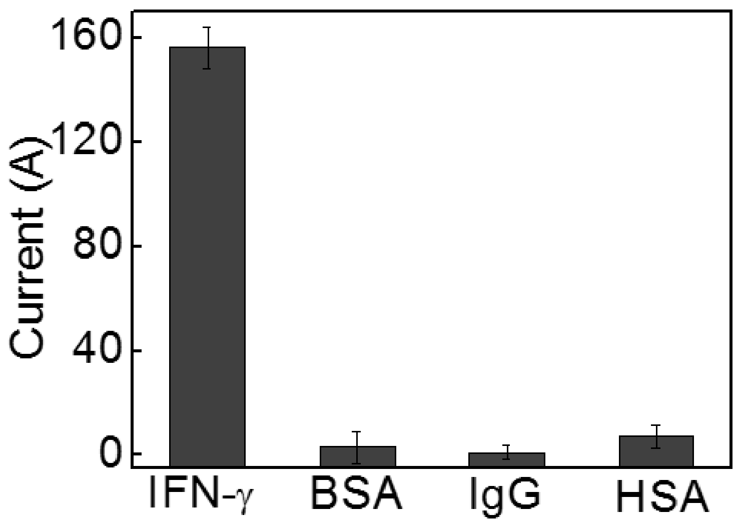

Figure S4. Selectivity of the sensor for the detection of 300 pM of IFN-γ compared with 3.0 nM of BSA, IgG and HSA.

Table S1. Sequences of oligonucleotides.

Name	Sequence (5'-3')
MB	CCCTGGGCTCAACCTAGGAATCGCGTTGGTTGTGTTGGGTGTTGTG TGCGATTCCT-SH-3'
H_1	GCGATTCCTAGGTTGAGCCCAGGGCACAGTCCCTGGGCTCAACCTAGG
H_2	CCCTGGGCTGAACCTAGGAATCGCCCTAGGTTGAGCCCAGGGACTGTG

Table S2. The analytical performance of our method compared to other methods.

Sensing element	Linear range	Detection limit	References
Graphene	--------	83 pM	4
Enzyme	0.5-300 nM	0.3 nM	7
Graphene	0.1 to 0.7 pM	65 fM	8
Iridium complex	1-300 nM	0.12 nM	28
streptavidin-incorporated aptamer	0.3-33 nM	33 pM	29
Graphene	50 to 0.01 ng mL^{-1}	0.003 ng mL^{-1}	30
Iridium complex	50 fM to 3.0 pM	16.3 fM	Our method

Table S3. Recovery experiments for IFN-γ in human serum.

Sample	Added (pM)	Found (pM)	Recovery %	RSD %
1	0.1	0.098	98	4.06
2	0.5	0.483	96.6	3.81
3	1.0	1.081	108.1	2.05
4	3.0	2.936	97.9	1.87
